# The synergy of serum SFRP5 levels and the TyG index in predicting coronary artery disease and prognosing major adverse cardiovascular events

**DOI:** 10.1186/s12944-023-01965-2

**Published:** 2023-11-13

**Authors:** Lin Jia, Shimei Shang, Yu Yang, Jian Zhang, Xianhe Lin

**Affiliations:** 1 Department of Cardiology, The First Affiliated Hospital of Anhui Medical University, Hefei city, Anhui province 230022 P.R. China; 2 Anhui Medical University, Hefei city, Anhui province 230032 P.R. China

**Keywords:** CAD, SFRP5, TyG index, MACE

## Abstract

**Background and aims:**

Secreted frizzled-related protein 5 (SFRP5) is a member of the SFRP family that is known for its potent anti-inflammatory properties. Nevertheless, little is known regarding the relevance of SFRP5 in coronary artery disease (CAD). The current study examined the correlation between serum levels of SFRP5 and the triglyceride-glucose (TyG) index in patients who underwent coronary angiography (CAG) as a component of cardiovascular assessment and for the purpose of prognosis evaluation.

**Methods:**

A total of 310 hospitalized patients were enrolled in this study between May 2021 and March 2022 and were divided into three groups based on their CAG results and SYNTAX (synergy between PCI with TAXUS drug-eluting stent and cardiac surgery) scores: the control group, mild lesion group, and moderate-severe lesion group. Univariate and multivariate analyses were employed to investigate the relationships between changes in patients and clinical variables. To investigate the impact of the TyG index and serum SFRP5 levels on the occurrence of major adverse cardiovascular events (MACEs), Kaplan‒Meier curves were plotted. Serum SFRP5 levels were measured utilizing an enzyme-linked immunosorbent assay (ELISA) kit.

**Results:**

The serum SFRP5 levels significantly decreased with the increasing severity and complexity of CAD, while the TyG index significantly increased (*P* < 0.001). Moreover, a significant negative correlation was observed between the serum SFRP5 levels and the TyG index (r = -0.312, *P* < 0.001). SFRP5 exerts a protective role in different groups of patients. The area under the receiver operating characteristic (ROC) curve indicated that an SFRP5 concentration > 115.58 pg/mL was the best predictive value for CAD (OR:0.87, *P* < 0.001). MACEs were significantly associated with serum SFRP5 levels and the TyG index, as indicated by both univariate and multivariate Cox regression analyses (*P* < 0.001). Furthermore, Kaplan‒Meier analysis indicated that as the TyG index decreased and SFRP5 levels increased, the occurrence of MACEs decreased (*P* < 0.001). Patients with a concentration of SFRP5 > 115.58 pg/mL and a TyG index < 8.49 exhibited a better prognosis for avoiding MACEs (*P* < 0.001).

**Conclusion:**

These results suggest that the collaboration between serum SFRP5 levels and the TyG index holds promise in predicting CAD and its prognosis.

## Introduction

Coronary atherosclerotic disease and its associated complications are substantial contributors to global mortality and disability [1]. Traditional diagnostic modalities for CAD evaluation include invasive techniques such as selective CAG intravascular ultrasound and optical coherence tomography, all of which are invasive and incur significant costs, thereby constraining their clinical utility [1, 2]. Consequently, there is an urgent need for a practical, low-risk, and cost-effective approach for predicting the occurrence and progression of CAD, as well as for assessing the extent of coronary artery lesions, given the pronounced clinical significance of this pursuit. SFRP5 is a member of the secretory glycoprotein SFRP family that is involved in both insulin resistance and inflammation [3]. Remarkably, SFRP5 exhibits cardioprotective properties, making it a promising candidate as an innovative biomarker for prognosticating the progression of cardiovascular disease [4–6]. Moreover, the TyG index has gained prominence as a straightforward measure for assessing insulin resistance [7].

This study aims to investigate the correlation between serum SFRP5 levels and the severity of CAD, as well as their synergistic effect with the TyG index. It is postulated that this synergy is attributed to the role of SFRP5 in regulating inflammatory responses and insulin resistance, while the TyG index directly reflects insulin resistance status and lipid health, creating a complementary relationship. This study, for the first time, combines serum SFRP5 levels and the TyG index for predicting CAD severity and prognosis of MACE. The aim is to identify high-risk patients more accurately through this unique approach and provide them with more personalized and precise medical interventions.

## Patients and methods

### Research population

This study was a single-center observational cohort study involving a total of 310 patients who underwent CAG at the Department of Cardiology, First Affiliated Hospital of Anhui Medical University, between May 2021 and March 2022, following predefined inclusion criteria. Case inclusion criteria: (1) meeting the diagnostic criteria established by the United States College of Cardiology Heart Association in 2020; (2) not recently taking drugs that affect SFRP5 levels, such as metformin, nonsteroidal anti-inflammatory drugs (NSAIDs), and medications used for obesity management; (3) Providing the informed consent agreement to participate and approval from the medical ethics committee. Exclusion criteria: (1) patients with congenital heart disease, severe heart failure, heart valve disease, myocarditis, pericardial diseases and other heart diseases; (2) patients with diseases that affect the test parameters, such as: serious infectious diseases and autoimmune diseases; and (3) patients with severe liver and renal insufficiency and abnormal coagulation function.

This study received approval from the institutional ethics committee at The First Affiliated Hospital of Anhui Medical University, which adhered to the principles outlined in the Declaration of Helsinki. Every patient provided written informed consent to participate, and precautions were implemented to safeguard the confidentiality of their identities.

### Data collection

Standard laboratory methods were employed to measure a range of parameters in the clinical laboratory of the First Affiliated Hospital of Anhui Medical University. These parameters included routine blood tests and D-dimer (D-D), fibrinogen (FIB), lactate dehydrogenase (LDH), creatine kinase-MB (CK-MB), glomerular filtration rate (eGFR), uric acid (UA), serum creatinine (Scr), fasting blood glucose (FBG) levels and glycated hemoglobin (HbA1c) levels. Plasma concentrations of total cholesterol (TC), triglyceride (TG), high-density lipoprotein cholesterol (HDL-C), low-density lipoprotein cholesterol (LDL-C), non-HDL-cholesterol (n-HDL-C), very low-density lipoprotein cholesterol (VLDL-C), apolipoprotein A-1 (ApoA-1), apolipoprotein B (ApoB), and lipoprotein(a) (Lp(a)) were measured using an automatic biochemistry analyzer (Hitachi 7150, Tokyo, Japan), following the manufacturer’s immunoturbidimetry method guidelines. Fasting venous blood was collected from the study subjects before CAG, and the supernatant was extracted for testing after centrifugation. The supernatant was divided into 2 parts, sub-packaged in EP tubes, and they were stored at -80 °C. An ELISA was utilized to determine the serum levels of SFRP5. The ELISA kit was procured from Shanghai Aibo Biotechnology Company and utilized in strict adherence to the provided reagent instructions. The severity of CAD was evaluated using the results obtained from CAG and SYNTAX scores. From the electronic medical record management system of the First Affiliated Hospital of Anhui Medical University, a range of data, including vital signs, past medical history, smoking and drinking habits, laboratory test results, electrocardiogram data, and other variables were retrieved.

### Definition of risk factors

The selection of variables primarily encompasses risk factors for CAD. BMI was determined by applying the following formula: body mass index (BMI) = weight (kg)/height^2^ (m^2^). The calculation for pulse pressure was as follows: pulse pressure (mmHg) = systolic blood pressure (SBP) (mmHg) - diastolic blood pressure (DBP) (mmHg). The TyG index was computed using the following formula: Ln [fasting triglycerides (mg/dL) × fasting blood glucose (mg/dL)/2] [8]. Hypertension was determined by meeting one of the following criteria: (1) currently prescribed antihypertensive medication, or (2) three consecutive resting blood pressure measurements showing an SBP ≥ 140 mmHg or a DBP ≥ 90 mmHg. The diagnosis of diabetes mellitus (DM) was established through the application of one of the following criteria: (1) a conclusive diagnosis ascertained by a medical professional, (2) persistent and prolonged use of medications prescribed for diabetes, or (3) meeting predetermined criteria, which include FBG levels ≥ 7.0 mmol/L, 2-hour postprandial blood glucose levels ≥ 11.1 mmol/L, or random blood glucose levels ≥ 11.1 mmol/L, as determined by an oral glucose tolerance test. The calculation of the neutrophil-to-lymphocyte ratio (NLR) involved dividing the neutrophil count (*10^9/L) by the lymphocyte count (*10^9/L). The patient’s medical history related to drinking and smoking was initially obtained from patient-provided information and subsequently confirmed through relevant laboratory examinations.

### Angiographic analyses

The SYNTAX score is a risk stratification tool used for evaluating CAD based on the anatomical characteristics of coronary artery lesions. It provides a quantitative assessment of coronary lesion complexity by considering factors such as lesion location, severity, branching patterns, and calcification. This scoring system is essential for comparing treatment outcomes between coronary artery bypass grafting (CABG) and percutaneous coronary intervention (PCI) in patients with left main CAD or triple-vessel disease (3VD). Depending on the SYNTAX score range, the severity of coronary artery stenosis was categorized as follows: (1) mild lesion: ≤22 points; (2) moderate lesion: 23–32 points; and (3) severe lesion: ≥33 points [9]. The coronary angiograms were independently analyzed by two highly experienced interventional cardiologists, who were blinded to the patients’ clinical data. Subsequently, the average of their assessments was computed.

### Statistical analysis

Statistical analysis was carried out using SPSS 26.0 software (IBM Corp, Armonk, NY, USA), and GraphPad Prism 9.0.2 (GraphPad Software, San Diego, CA, USA) was employed for generating graphs and charts. To evaluate the data distribution, the Kolmogorov‒Smirnov test for normality was used. Continuous variables were reported as either means with standard deviations or medians with interquartile ranges, depending on the outcome of the normality test. For continuous variables with a normal distribution, analysis of variance (ANOVA) was performed to examine group differences. In contrast, for nonnormally distributed continuous variables, the Kruskal‒Wallis H test was performed to assess variations between groups. Categorical variables are presented as counts (percentages), and group differences were evaluated using either the chi-squared test or Fisher’s exact test. Spearman’s correlation coefficient was employed to investigate the relationships between individual independent variables and SFRP5. Logistic regression models were employed to assess the association between each index and the severity of the disease. ROC curves were created to evaluate the diagnostic accuracy of each index for CAD. Patients were stratified into two groups based on the best predictive values, which were determined by sensitivity and specificity calculations. Univariate Cox regression analysis was performed to predict MACEs based on different independent variables. The multivariate Cox regression analysis incorporated independent variables related to lipid metabolism. Finally, Kaplan–Meier curves were utilized to illustrate the MACE risk in both the reduced and normal SFRP5 groups.

### MACE

Qualified health care professionals conducted patient monitoring through either clinical visits or telephone communication. Follow-up was performed until July 23, 2023, with a median follow-up duration of 17 months (interquartile range: 13 to 17). MACEs included cardiac mortality, nonfatal myocardial infarction, target lesion revascularization, rehospitalization for stroke treatment, and episodes of unstable or worsening angina [10].

## Results

### Baseline clinical characteristics

Patients were categorized into three groups based on their CAG results and SYNTAX scores: a control group (n = 50), a mild lesion group (< 23 points, n = 175) and a moderate-severe lesion group (≥ 23 points, n = 85). Table 1 outlines the baseline clinical characteristics observed within the study population. No significant differences were observed among the groups (*P* > 0.001) regarding age, sex, BMI, smoking history, drinking history, hypertension, n-HDL-c, VLDL-C, Scr, and eGFR assay results. Lp(a) and UA levels significantly differed only between the control group and the moderate-severe lesion group. D-D levels significantly differed only between the mild group and the moderate-severe lesion group. LDH and CK-MB values differed significantly between the control group and the mild lesion group and between the mild lesion group and the moderate-severe lesion group. The levels of ApoA1 and ApoB were significantly different between the control group and the mild and moderate-severe lesion groups, whereas no significant difference was observed between the mild group and the moderate-severe group. There was a substantial increase in the concentrations of TC, TG, LDL-C, SFRP5, HbA1c and TyG, and this increase was statistically significant across all groups. In contrast, both HDL-C and EF levels were significantly decreased.

**Table 1:** Baseline clinical characteristics

Variables	Control	Mild lesion	Moderate-Severe lesion	X²/F	*P*
	(n = 50)	(n = 175)	(n = 85)		
**Demographic data**					
Age (years)	60.92 ± 10.94	61.65 ± 12.16	64.45 ± 10.00	2.16	0.117
Male sex, n (%)	27(54.00%)	126(72.00%)	58(68.20%)	7.59	0.055
BMI (kg/m²)	24.58 ± 3.87	25.10 ± 3.69	24.78 ± 2.80	0.53	0.592
**Medical history**					
Smoking, n (%)	19(38.00%)	81(46.30%)	40(47.10%)	1.25	0.536
Drinking, n (%)	12(24.00%)	55(31.40%)	22(25.90%)	1.51	0.471
Hypertension, n (%)	21(42.00%)	96(54.90%)	53(62.40%)	5.27	0.072
T2DM, n (%)	10(20.00%)	32(18.30%)^a^	29(34.10%)^b^	8.41	< 0.001
**Laboratory measurement**					
TC (mmol/L)	4.61 ± 0.89	4.82 ± 1.02^a^	5.47 ± 1.23^ab^	12.83	< 0.001
TG (mmol/L)	0.79(0.56,0.99)	1.35(1.05,1.70)^a^	2.28(1.82,3.01)^ab^	155.24	< 0.001
HDL-C (mmol/L)	2.17 ± 0.64	1.22 ± 0.34^a^	0.93 ± 0.32^ab^	87.10	< 0.001
LDL-C (mmol/L)	1.56(1.22,2.14)	2.87(2.36,3.54)^a^	4.06(2.70,4.66)^ab^	90.34	< 0.001
nHDL (mmol/L)	2.90(2.41,3.75)	2.85(2.27,3.53)	2.90(2.23,3.86)	1.38	0.501
VLDL (mmol/L)	0.49(0.39,0.77)	0.54(0.39,0.79)	0.52(0.39,0.86)	1.06	0.587
ApoA1 (g/L)	1.34 ± 0.27	1.24 ± 0.19^a^	1.23 ± 0.19^a^	4.39	< 0.001
ApoB (g/L)	0.69 ± 0.22	0.85 ± 0.28^a^	0.93 ± 0.33^a^	10.19	< 0.001
SFRP5 (pg/mL)	123.24 ± 13.74	102.75 ± 16.34^a^	87.11 ± 12.96^ab^	91.21	< 0.001
Lp-a (mmol/L)	92.00(34.50,223.50)	145.00(66.00,315.00)	190.00(72.00,369.50)^a^	7.48	< 0.001
D-D (mg/L)	0.25(0.16,0.41)	0.22(0.15,0.39)	0.32(0.23,0.51)^b^	20.25	< 0.001
FIB (g/L)	3.06(2.54,3.58)	2.98(2.59,3.48)	3.23(2.69,3.82)^ab^	7.51	< 0.001
LDH (U/L)	204.00(174.50,227.00)	178.00(158.50,207.25)^a^	194.50(161.75,229.25)^b^	10.93	< 0.001
CK-MB (U/L)	11.00(8.00,12.00)	12.00(9.75,16.00)^a^	14.00(11.00,21.25)^b^	21.93	< 0.001
eGFR (ml/min)	96.00(82.50,109.00)	94.50(81.00,106.25)	94.50(81.25,103.00)	0.78	0.679
UA (mmol/L)	322.22 ± 93.78	358.32 ± 95.91	370.38 ± 108.55^a^	3.84	< 0.001
Scr (umol/L)	71.10(56.65,79.55)	74.15(61.55,86.18)	73.65(63.28,85.23)	2.36	0.307
FBG (mmol/L)	4.89(4.56,5.43)	5.25(4.74,5.79)	5.84(5.10,7.21)^ab^	36.86	< 0.001
HbA1c (%)	4.90(4.40,5.45)	5.40(4.90,6.30)^a^	6.00(5.30–6.75)^ab^	38.51	< 0.001
TyG Index	8.19 ± 0.89	8.67 ± 0.58^a^	9.34 ± 0.54^ab^	59.05	< 0.001
EF (%)	61.00(59.00,63.00)	60.00(57.00,63.00)	58.50(54.00,64.00)^ab^	12.59	< 0.001
LVD (cm)	4.63(4.47,5.06)	4.88(4.61,5.18)	4.98(4.59,5.30)^a^	6.56	< 0.001

Values are presented as the median (interquartile range), n (%), or the mean ± standard deviation. *P*-values were calculated using ANOVA, the chi-square test, the Kruskal-Wallis test, or Fisher’s test. A significance level of *P* < 0.05 was considered statistically significant (^a^: compared to the control group, *P* < 0.05; ^b^: compared to the mild lesion group, *P* < 0.05)

### SFRP5 and TyG index

**Fig. 1:**
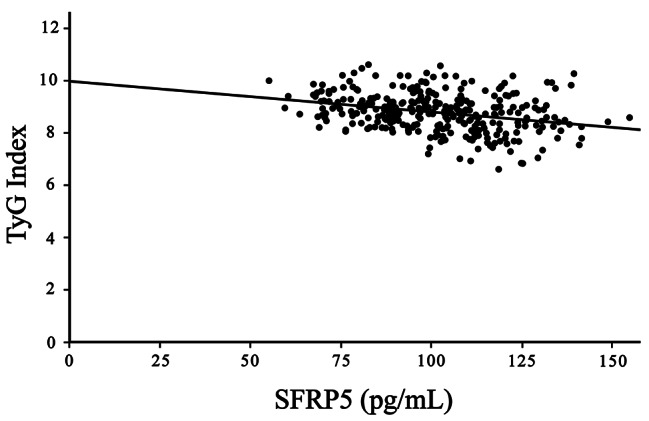
Scatter diagram showing the correlation between serum SFRP5 levels and the TyG index

Table 2 presents the correlation analysis among serum SFRP5 levels, the TyG index, and various variables within the patient cohort. Notably, serum SFRP5 levels were positively correlated with HDL-C. However, they were negatively correlated with TG, LDL-C, and HbA1c levels and SYNTAX scores, all of which were statistically significant (*P* < 0.001), and negatively correlated with FBG (r=-0.160, *P* = 0.005). Conversely, the TyG index was positively correlated with LDL-C, TG, FBG, and HbA1c levels and SYNTAX scores and was negatively correlated with HDL-C levels (*P* < 0.001). Importantly, the analysis revealed a significant inverse relationship between serum SFRP5 levels and the TyG index (r= -0.312, *P* < 0.001) (Fig. 1).

**Table 2:** The correlation between serum SFRP5 levels and the TyG index

Variables	SFRP5		TyG	
	r	*P*	r	*P*
SFRP5			-0.312	< 0.001
TyG	-0.312	< 0.001		
TG	-0.504	< 0.001	0.657	< 0.001
LDL-C	-0.364	< 0.001	0.273	< 0.001
HDL-C	0.476	< 0.001	-0.377	< 0.001
FBG	-0.160	0.005	0.379	< 0.001
HbA1c	-0.209	< 0.001	0.257	< 0.001
SYNTAX scores	-0.565	< 0.001	0.585	< 0.001

### Multiple logistic regression analysis of risk factors of CAD

**Fig. 2:**
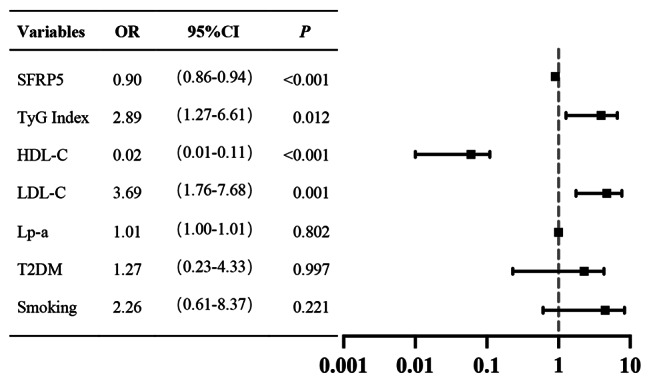
Forest plot of the results of the multivariate logistic regression model exploring the association between plasma SFRP5 levels and other related factors in patients with CHD.

Figure 2 displays the results of multiple logistic regression analysis, examining the association between serum SFRP5 levels and various relevant variables in patients who enrolled in this research. The forest plot presents the results of the multivariate logistic regression model that examined the relationship between serum SFRP5 levels and various other factors. In the analysis, patients with CAD were considered the dependent variable, while SFRP5, TyG index, LDL-C, and HDL-C were considered independent variables. The logistic multivariate regression analysis revealed that the TyG index and LDL-C were independent risk factors for CAD, while SFRP5 and HDL-C were protective factors for CAD after adjusting for other confounding factors (*P* < 0.05). These findings suggest that an elevated TyG index, along with increased LDL-C levels, is associated with an increased risk of CAD. Conversely, higher levels of SFRP5 and HDL-C are associated with a reduced risk of CAD.

### Diagnostic value of serum SFRP5 levels and the TyG index

**Fig. 3:**
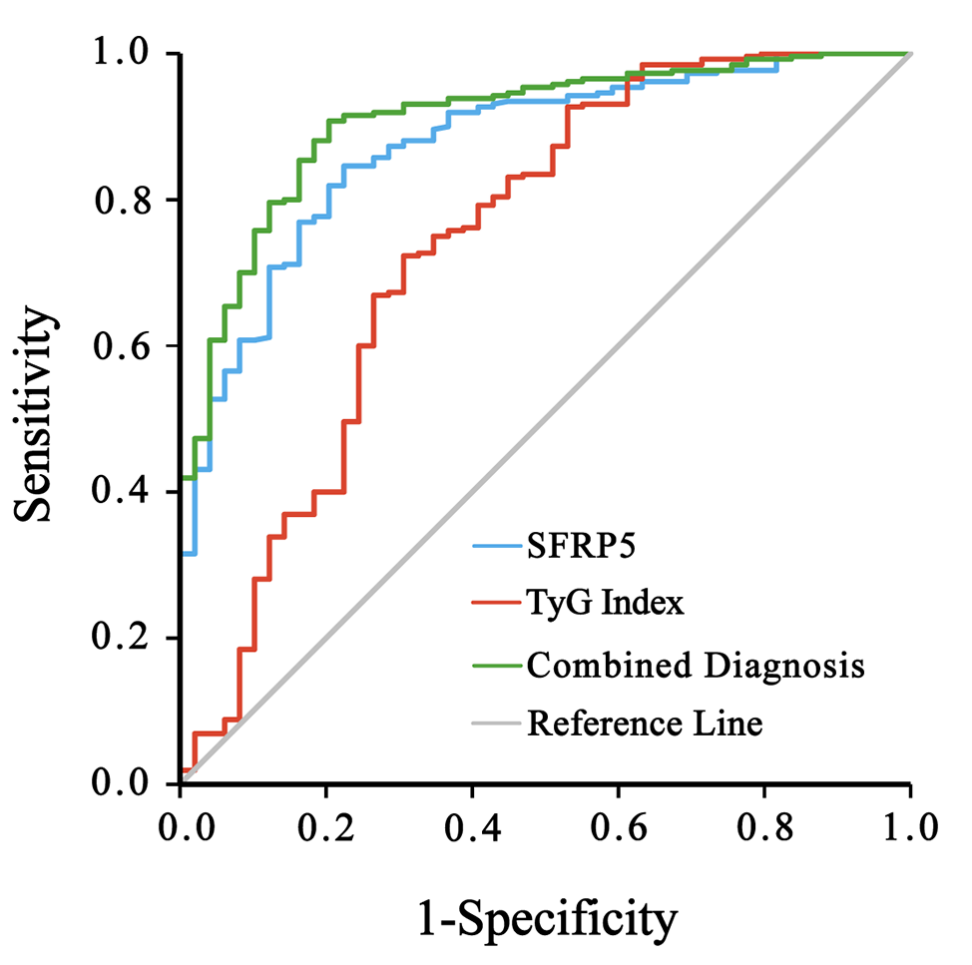
Diagnostic value of plasma SFRP5 levels and the TyG index in ROC curve

The diagnostic value of serum SFRP5 levels and the TyG index was illustrated in the ROC curve for the occurrence of CAD. The optimal cutoff value for the serum SFRP5 concentration was determined to be 115.58 pg/mL. At this threshold, the sensitivity, specificity, and AUC for predicting coronary heart disease were 84.6%, 77.6% and 0.87 [95% CI (0.82,0.92), *P* < 0.001], respectively. Similarly, the optimal cutoff value for the TyG index was determined to be 8.49, with corresponding sensitivity, specificity, and AUC values of 72.3%, 69.4%, and 0.74 [95% CI (0.65,0.83), *P* < 0.001], respectively. Notably, when both serum SFRP5 levels and the TyG index were considered together, the combination exhibited improved predictive performance, with corresponding sensitivity, specificity, and AUC values of 90.8%, 79.4%, and 0.91 for predicting the occurrence of CAD [95% CI (0.87, 0.95), *P* < 0.001] (Fig. 3 & Table 3).

**Table 3:** Diagnostic value of serum SFRP5 levels and the TyG index in ROC curve

	AUC	Optimal Cut-Off Value	Sensitivity	Specificity	95%CI		*P*
——SFRP5:	0.87	115.58pg/ml	84.60%	77.60%	0.82	0.92	< 0.001
——TyG Index:	0.74	8.49	72.30%	69.40%	0.65	0.83	< 0.001
——Combined Diagnosis:	0.91		90.80%	79.40%	0.87	0.95	< 0.001

### The SFRP5 levels, TyG index, and MACEs

**Fig. 4:**
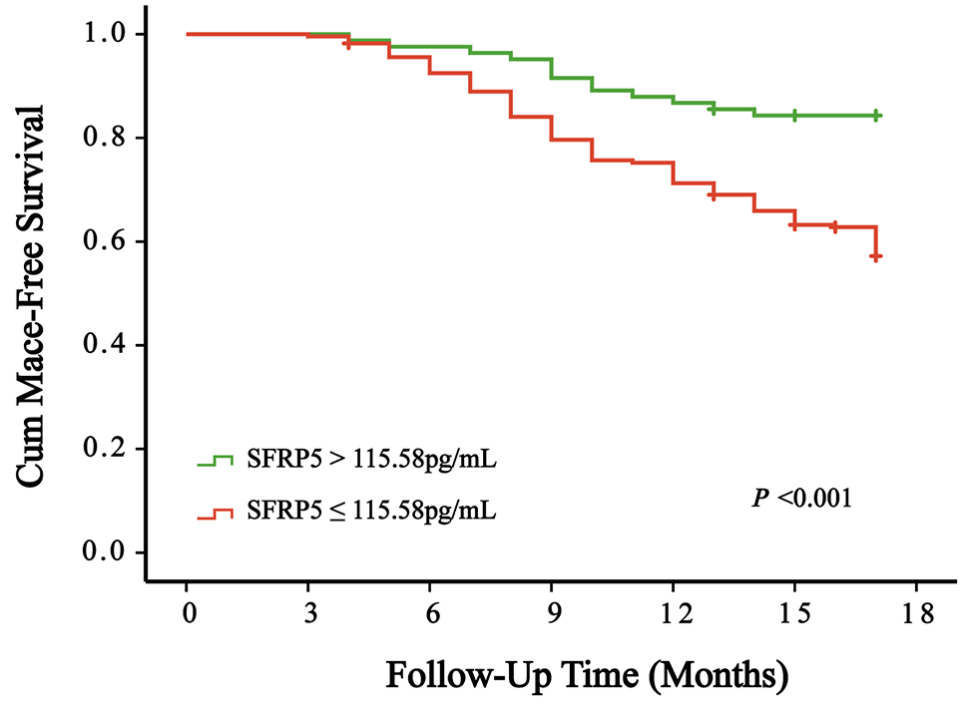
Kaplan‒Meier curves for MACEs of SFRP5.

**Fig. 5:**
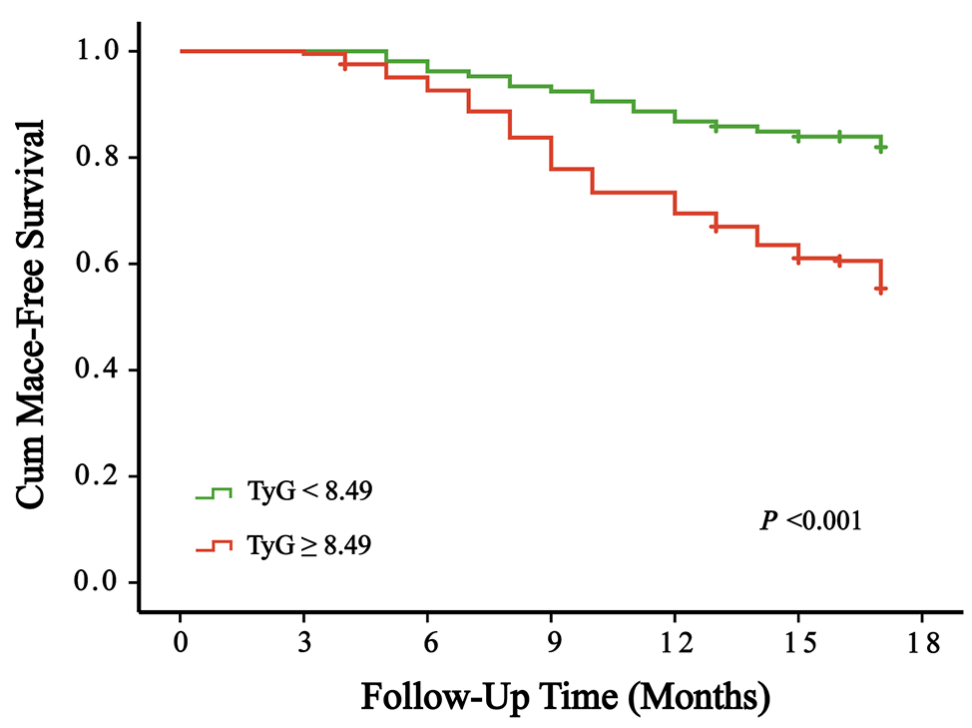
Kaplan‒Meier curves for MACEs of the TyG index

**Fig. 6:**
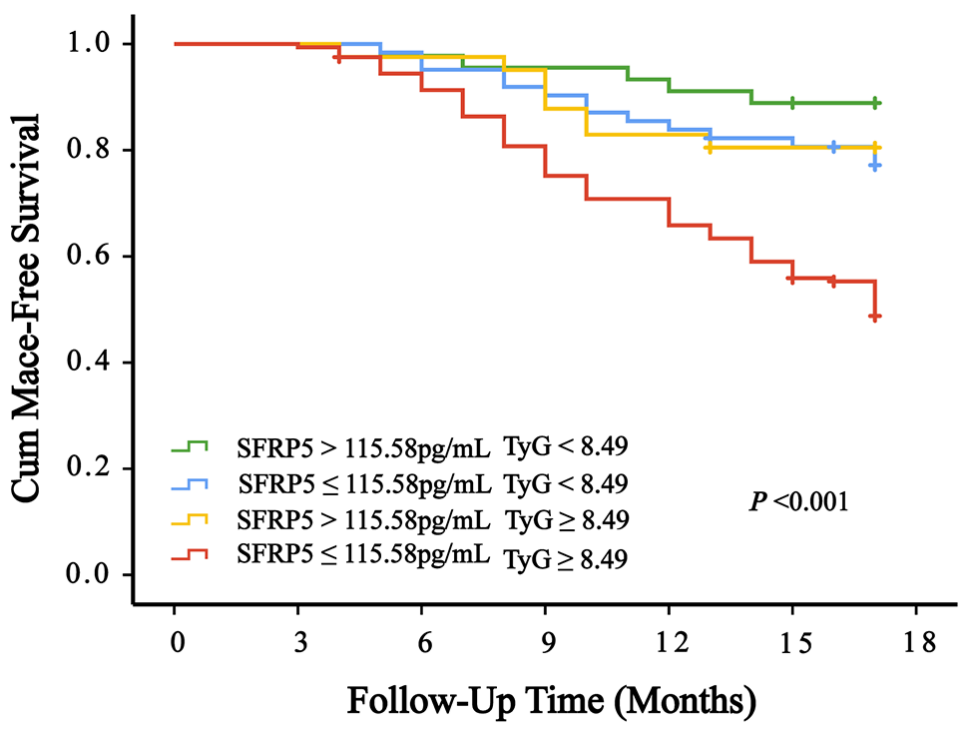
Kaplan‒Meier curves for MACEs among SFRP5 and the TyG index

Table 4 provides the results of the univariate Cox regression analysis, demonstrating the independent roles of variables as predictors for clinical endpoint events. In this analysis, statistically significant independent associations were observed for surgery time, sex, LVD, EF, SFRP5, TyG index, TG, TC, HDL-C, LDL-C, ApoB, Lp(a), FDP, FIB, D-D, LDH, CK-MB, UA, r, BNP, ALT, and AST (*P* < 0.05). Using the stepwise forward method, the multivariate Cox regression analysis revealed significant associations (Table 5) for SFRP5 (*P* = 0.01), TyG index (*P* = 0.001), and LDL-C (*P* = 0.043). The Kaplan‒Meier analysis, assessing event-free survival, demonstrated a clear trend. As the TyG index increased, the incidence of MACE increased and SFRP5 levels decreased (*P* < 0.001) (Fig. 4 & Fig. 5). Patients with a concentration of SFRP5 > 115.58 pg/mL and a TyG index < 8.49 exhibit a better prognosis for avoiding MACEs (*P* < 0.001) (Fig. 6).

**Table 4:** Univariate Cox regression analysis of MACE

Variables	OR	95%CI		*P*
		Lower	Upper	
Surgery time	1.011	1.006	1.015	< 0.001
Age	1.014	0.998	1.031	0.093
Sex	1.701	1.088	2.659	0.020
BMI	0.956	0.905	1.011	0.115
Smoking	1.401	0.962	2.039	0.079
Drinking	1.185	0.790	1.777	0.412
T2DM	1.317	0.861	2.014	0.204
Hypertension	0.952	0.653	1.388	0.799
Previous PCI	0.864	0.537	1.391	0.547
Previous CAG	1.299	0.883	1.911	0.184
LVD	1.498	1.143	1.964	0.003
EF	0.940	0.920	0.961	< 0.001
SFRP5	0.973	0.963	0.983	< 0.001
TyG	1.869	1.445	2.418	< 0.001
TG	1.529	1.304	1.792	< 0.001
TC	1.302	1.098	1.545	0.002
HDL-C	0.431	0.285	0.653	< 0.001
LDL-C	1.458	1.246	1.707	< 0.001
VLDL-C	1.034	0.717	1.491	0.859
ApoA-1	0.547	0.206	1.448	0.224
ApoB	2.752	1.551	4.883	0.001
Lp(a)	1.001	1.000	1.001	0.021
FDP	1.042	1.009	1.076	0.011
FIB	1.063	1.012	1.117	0.015
D-D	1.147	1.041	1.264	0.006
LDH	1.001	1.001	1.002	< 0.001
CK-MB	1.010	1.003	1.017	0.004
eGFR	0.993	0.984	1.002	0.113
UA	1.004	1.002	1.006	< 0.001
Scr	1.007	1.001	1.013	0.024
BNP	1.002	1.001	1.002	< 0.001
ALT	1.007	1.001	1.012	0.020
AST	1.004	1.002	1.006	< 0.001
ALP	1.003	0.997	1.009	0.299

**Table 5:** Multivariate Cox regression analysis of MACE

Variables	OR	95% CI		*P*
		Lower	Upper	
SFRP5	0.984	0.973	0.996	0.010
TyG Index	1.816	1.271	2.596	0.001
TC	0.688	0.415	1.140	0.147
HDL-C	1.323	0.658	2.663	0.432
LDL-C	1.688	1.016	2.804	0.433
Lp(a)	1.000	1.000	1.001	0.369

## Discussion

This study was designed to explore the correlation between serum SFRP5 levels and the TyG index, as well as their collective influence on MACEs in individuals diagnosed with CAD. There were several main findings of this study. (1) Serum SFRP5 levels were significantly negatively correlated with the TyG index. (2) Assessing the combination of both SFRP5 and the TyG index demonstrates superior predictive efficacy for CAD compared to evaluating either marker alone. (3) Adding SFRP5 to the existing risk prediction model could improve MACE prediction in patients with CAD. Hyperlipidemia is widely acknowledged as an independent risk factor for coronary atherosclerosis, contributing to the increased complexity and severity of coronary artery lesions and thereby significantly affecting patient prognosis [11, 12].

SFRP5 is a member of the SFRP family, which is characterized by a cysteine-rich domain (CRD) in the extracellular region, and it is secreted by adipocytes. This domain binds to Wnt proteins and effectively inhibits the transmission of Wnt signals. SFRP5 plays a crucial role as a modulator in various cellular processes, including but not limited to cell proliferation, differentiation, migration, and apoptosis, owing to its inhibitory function [13–15]. In obese animal models, a significant reduction in SFRP5 expression levels has been observed, and this deficiency may be strongly associated with insulin resistance and adipose tissue inflammation [16–18]. Noriyuki Ouchi et al. discovered that SFRP5-deficient mice exhibit no discernible alterations under a standard diet but exhibit pronounced inflammation in their adipose tissue and systemic metabolic dysfunction when subjected to a high-calorie diet. Conversely, the administration of SFRP5 in mouse models of obesity and diabetes yields contrasting outcomes, enhancing metabolic functionality and mitigating inflammation within adipose tissue [16]. This discovery suggests that SFRP5 within adipose tissue holds promise as a potential therapeutic target for addressing glucose homeostasis abnormalities linked to obesity.

In the present investigation, a trend that contradicts the findings of Lu et al. [19] emerged, as an inverse relationship between SFRP5 levels and both FBG and HbA1c levels was observed. The majority of studies suggest a potential positive association between SFRP5 and insulin sensitivity, promoting more effective cellular responses to insulin and thus contributing to blood glucose regulation [20, 21]. It is essential to acknowledge that there are also studies indicating a positive correlation between SFRP5 and insulin resistance, along with other metabolic abnormalities [19, 22]. These discrepancies underscore the idea that the mechanism through which SFRP5 impacts inflammation and insulin resistance may be influenced by specific medications, individual patient characteristics, and the overall metabolic context. Therefore, further research is needed to further investigate these mechanisms and gain a more comprehensive understanding of the role of SFRP5 in cardiovascular health and metabolic diseases. Recent investigations have also demonstrated that SFRP5 enhances the stability of carotid plaques by reducing proinflammatory cytokine levels in macrophages. These cumulative findings further underscore the potential therapeutic implications of SFRP5 in the management of cardiovascular and systemic diseases [23, 24]. Tong et al. have demonstrated that low SFRP5 levels and high Wnt5a levels are associated with the presence of CAD [25]. However, Du et al. presented a further perspective. They observed that in patients with ST-segment elevation myocardial infarction (STEMI) upon admission, serum SFRP5 levels initially increase and subsequently decline after timely reperfusion therapy. This acute elevation of SFRP5 levels upon admission is associated with early improvement in left ventricular function and is independently correlated with peak cTnI (cardiac troponin I) levels and baseline cardiac function [15]. These findings imply that the initial surge in SFRP5 levels upon admission may be indicative of a stress response, possibly representing the body’s physiological adaptation to myocardial infarction. As reperfusion therapy advances, the subsequent decline in SFRP5 levels may signify enhanced cardiac function and the repair of myocardial injury. This phenomenon suggests a potential role for SFRP5 in predicting or assessing cardiac function recovery throughout the clinical trajectory following myocardial infarction.

This study also incorporated the TyG index. The TyG index has emerged in recent years as a straightforward and practical indicator for assessing insulin resistance (IR) [26]. In the present study, a significant correlation among serum SFRP5 levels, the TyG index, and the severity of coronary stenosis in CAD was observed. Moreover, the outcomes unveil a notable decrease in the SFRP5 concentration in high SYNTAX groups, suggesting that reduced levels of SFRP5 exert a detrimental effect on the pathology of atherosclerosis. The results of the present study show that serum SFRP5 levels are significantly decreased and negatively correlated with the severity of the condition, which aligns with numerous previous studies [27, 28]. These findings suggest that both serum SFRP5 levels and the TyG index could serve as valuable markers for evaluating lipid metabolism and atherosclerosis. Multivariate logistic regression analysis reveals that TyG serves as an independent risk factor for CAD, whereas SFRP5 plays a protective role in this context. The ROC curve analysis also indicated that SFRP5 has a significant predictive value for CAD. In this study, a combined analysis of SFRP5 and the TyG index was used to assess the diagnostic predictive value for CAD patients. There was a notable increase in diagnostic specificity and positive predictive value for CAD (sensitivity at 90.8%, specificity at 79.4%, AUC at 0.91). This underscores the advantage of the combined detection of these two indicators in accurately predicting the occurrence of CAD. Multifactorial Cox regression analysis suggested that SFRP5 and TyG are both predictive factors for MACEs.

Thus, patients who underwent CAG could be categorized into two groups based on their SFRP5 levels, confirming its suitability for both diagnostic and long-term prognostic purposes. After a follow-up period of 17 months, with an interquartile range of 13 to 17 months, Kaplan‒Meier curves were constructed. From the curves, it becomes evident that with the extension of follow-up time, patients with lower serum SFRP5 concentrations and a higher TyG index have a greater risk of developing MACEs. Based on the cutoff values obtained from the ROC curve and the Kaplan‒Meier analysis, patients with SFRP5 concentrations > 115.58 pg/mL and a TyG index < 8.49 have the lowest risk of MACEs. These findings provide substantial evidence for the diagnosis and risk assessment of CAD. As a result, future clinical approaches aimed at managing CAD should thoroughly explore the roles of SFRP5 and the TyG index. Overall, this study underscores the importance of conducting further research to comprehensively grasp the clinical implications of SFRP5 and the TyG index in the context of CAD.

## Study strengths and limitations

This study presents novel findings on the correlation between serum SFRP5 levels and the TyG index in patients. It explores the variances in serum SFRP5 levels and the TyG index across different subgroups of CAD while investigating their association with coronary stenosis. The findings propose that the measurement of serum SFRP5 levels and the TyG index holds promise as potential markers to predict the occurrence and progression in patients with CAD, as well as evaluate coronary disease.

Nonetheless, it is crucial to acknowledge the limitations inherent in this clinical study. First, the study adopts a cross-sectional study conducted at a single center, which introduces potential bias. Furthermore, the limited sample size undermines the robustness of the results. Additionally, the absence of longer-term follow-up data on adverse cardiovascular events in CAD patients hampers our ability to conduct long-term prognosis assessments. Moreover, it is crucial to explore the underlying mechanisms of SFRP5 in regulating the progression of coronary heart disease. Comprehensive animal experiments are essential to provide insights into these mechanisms.

Consequently, it is necessary to conduct large-scale, multicenter, prospective studies, along with additional animal experiments, to validate the diagnostic, severity, and prognostic significance of SFRP5 and the TyG index in relation to CAD.

## Conclusion

This study has elucidated the critical association between serum SFRP5 levels and the TyG index and their pivotal roles in CAD. Through rigorous analysis, it was demonstrated that while higher levels of serum SFRP5 act as a protective factor, a higher TyG index is associated with an increased risk of CAD. The combined assessment of serum SFRP5 levels and the TyG index has been shown to enhance the predictive accuracy for CAD, surpassing the performance of each biomarker used independently. From a clinical perspective, this integrated approach ensures a more reliable and precise identification of at-risk patients. This facilitates timely and targeted interventions and aids clinicians in stratifying patients based on their risk, leading to a reduction in the incidence of MACEs. Future clinical practices may consider incorporating serum SFRP5 levels and the TyG index as standard elements of CAD risk assessment and management, ensuring that every patient receives the most informed and effective care possible.

## Data Availability

The datasets analyzed during the current study are not publicly available due to privacy protection but are available from the corresponding author upon reasonable request.

## Abbreviations

SFRP5: Secreted frizzled-related protein 5
TyG index: Triglyceride-glucose index
MACE: Major adverse cardiovascular events
CAD: Coronary artery disease
CAG: Coronary arteriography
SYNTAX: Synergy between percutaneous coronary intervention with taxus and cardiac surgery
IR: insulin resistance
ANOVA: Analysis of variance
ELISA: Enzyme-linked immunosorbent assay
T2DM: Type II diabetes mellitus
ELISA: Enzyme-linked immunosorbent assay
ROC: Receiver operating characteristic
ApoA-1: Apolipoprotein A-1
ApoB: Apolipoprotein B
Lp(a): Lipoprotein(a)
Scr: Serum creatinine
FBG: Fasting blood glucose
HbA1c: Glycated hemoglobin
n-HDL-C: non-HDL-cholesterol
BMI: Body mass index
UA: Uric acid
HDL-C: High-density lipoprotein cholesterol
LDL-C: Low-density lipoprotein cholesterol
VLDL-C: very low-density lipoprotein cholesterol
TG: Triglyceride
TC: Total cholesterol
LDH: Lactate dehydrogenase
FIB: Fibrinogen
D-D: D-Dimer assay
CK-MB: Creatine kinase-MB
eGFR: Estimated glomerular filtration rate
NLR: Neutrophil-to-lymphocyte ratio
AUC: Area under the curve
OR: Odds ratio
CI: Confidence interval
